# Entropy-Based CT Radiomics as an Imaging Marker of Hepatic Injury in COVID-19

**DOI:** 10.3390/diagnostics15182364

**Published:** 2025-09-17

**Authors:** Alin Iulian Feiereisz, George-Călin Oprinca, Victoria Birlutiu

**Affiliations:** 1 Faculty of Medicine, Lucian Blaga University of Sibiu, Str. Lucian Blaga, Nr. 2A, 550169 Sibiu, Romania; georgecalin.oprinca@ulbsibiu.ro (G.-C.O.); victoria.birlutiu@ulbsibiu.ro (V.B.); 2 Infectious Diseases Clinic, Academic Emergency Hospital Sibiu, 550245 Sibiu, Romania; 3 Department of Pathology, Academic Emergency Hospital Sibiu, 550245 Sibiu, Romania

**Keywords:** CT radiomics, texture analysis, first-order entropy, gray-level co-occurrence matrix (GLCM), hepatic imaging, COVID-19, 3D Slicer, open-source

## Abstract

**Background:** Hepatic involvement in COVID-19 is frequently observed, yet conventional CT imaging may fail to detect subtle parenchymal alterations. This study aimed to evaluate whether CT-based radiomic texture analysis can identify liver injury associated with SARS-CoV-2 infection. **Methods:** We retrospectively analyzed 41 patients with RT-PCR–confirmed moderate or severe COVID-19 pneumonia who underwent non-contrast thoracoabdominal CT during the acute phase and at follow-up. Liver volume, mean hepatic attenuation, liver-to-spleen attenuation ratio, and radiomic features including first-order and GLCM entropy were extracted using 3D Slicer version 5.6.2 and SlicerRadiomic Revision: 8426cdf. Hepatic injury was defined by elevated serum transaminases. Three additional patients with available liver histopathology were included for correlation with imaging findings. **Results:** Patients with biochemical liver injury demonstrated significantly higher hepatic entropy values in the acute phase compared to those without injury (first-order entropy: 1.63 vs. 1.48, *p* = 0.019; GLCM entropy: 3.12 vs. 2.83, *p* = 0.013). Entropy metrics were inversely correlated with hepatic attenuation at follow-up (GLCM r = −0.385, *p* = 0.013; first-order r = −0.346, *p* = 0.027), indicating possible progression to lower-density states. Ferritin showed a moderate positive correlation with entropy (r = 0.47, *p* = 0.0017). Histopathological examination revealed steatosis, hepatocellular injury, inflammatory infiltration, and vascular congestion, aligning with radiomic abnormalities. **Conclusions:** Entropy-based CT radiomics reflect microstructural liver alterations in COVID-19, supported by both biochemical and histopathological data. This approach may enhance the detection of hepatic injury beyond conventional imaging and could be explored in systemic infections.

## 1. Introduction

Hepatic involvement has emerged as one of the most prevalent extrapulmonary manifestations of SARS-CoV-2 infection, increasingly recognized not only through elevated liver enzymes but also through histopathological and imaging studies that suggest complex patterns of liver damage. In hospitalized COVID-19 patients, elevations in aminotransferases, particularly alanine aminotransferase (ALT) and aspartate aminotransferase (AST), are reported in up to 50% of cases, typically in the absence of overt hepatic failure, which often leads to their underestimation in clinical significance [1].

Histological examinations in patients with SARS-CoV-2 infection have revealed a broad spectrum of hepatic alterations. Steatosis appears frequently in both macrovesicular and microvesicular forms and is often accompanied by varying degrees of portal and lobular inflammation. Additional features such as sinusoidal congestion, centrilobular necrosis, and cholestatic changes have also been described. The diversity of these findings suggests that liver injury results from multiple converging mechanisms rather than a singular direct viral effect [2]. While the liver is not considered a major replication site, it expresses angiotensin-converting enzyme 2 (ACE2) receptors, primarily on cholangiocytes and to a lesser extent on hepatocytes, allowing for potential viral entry. Rare identification of viral particles within hepatocytes on electron microscopy, along with mitochondrial swelling, cytoplasmic vacuolation, and endoplasmic reticulum dilation, suggests the possibility of direct viral damage, although this remains inconsistently demonstrated [3].

Liver injury in the context of COVID-19 is believed to result primarily from a combination of indirect, multifactorial mechanisms rather than direct viral cytotoxicity. One of the central contributors is the systemic hyperinflammatory response triggered by SARS-CoV-2, which disrupts hepatocyte homeostasis and leads to activation of resident immune cells within the liver. This cascade promotes immune-mediated damage to the hepatic parenchyma, characterized by lobular inflammation and hepatocellular stress. In parallel, respiratory dysfunction associated with moderate to severe COVID-19 can result in hepatic hypoxia, ultimately causing centrilobular ischemic necrosis, a pattern commonly observed in hypoxic hepatitis [4].

Vascular injury further exacerbates hepatic compromise. Damage to the endothelium and the formation of microvascular thrombi impair intrahepatic blood flow, contributing to perfusion deficits and enhancing susceptibility to ischemic injury. These vascular disturbances not only limit oxygen delivery but may also potentiate inflammatory signaling and hepatocellular apoptosis. Another layer of complexity arises from the pharmacologic agents administered during the acute management of COVID-19. Therapeutic protocols frequently include antivirals, corticosteroids, and broad-spectrum antibiotics, all of which have known hepatotoxic potential. The cumulative hepatic burden of these drugs may not be clinically evident in patients with normal liver function but can significantly amplify injury in predisposed individuals. This vulnerability is especially relevant in patients with pre-existing liver disease. In particular, individuals with non-alcoholic fatty liver disease (NAFLD) may experience disproportionate hepatic stress due to a reduced functional reserve, underlying steatosis, and chronic low-grade inflammation. These patients are more susceptible to additional insults and may have a diminished capacity for hepatic recovery, making them more likely to develop persistent liver dysfunction or progression to steatohepatitis following COVID-19 [5].

Although biochemical markers of liver injury are frequently elevated in patients with COVID-19, imaging findings on computed tomography are often inconsistent and lack diagnostic specificity. Non-contrast CT scans may show decreased hepatic attenuation or a reduced liver to spleen attenuation ratio, both of which are commonly associated with hepatic steatosis. In the context of COVID-19, however, these changes may also reflect inflammation, cellular edema, or vascular congestion, rather than true fat accumulation [6].

Attenuation values can remain low even after clinical recovery and normalization of liver enzymes, raising concern that conventional CT may overlook ongoing parenchymal injury. In many cases, the presence of steatosis may conceal more subtle inflammatory or ischemic changes within the liver, which are not readily detectable using standard density-based evaluation. This limitation reduces the sensitivity of conventional imaging for identifying early or mixed-pattern liver damage in patients recovering from COVID-19 [7].

Radiomics has emerged as a powerful extension of medical imaging analysis, offering the ability to extract high-throughput, quantitative descriptors from standard imaging datasets. Unlike conventional radiologic interpretation, which is largely qualitative and observer-dependent, radiomics enables objective assessment of tissue characteristics by analyzing pixel- or voxel-level data patterns. This approach captures subtle variations in texture, intensity, shape, and spatial arrangement that are often imperceptible on visual inspection, yet may reflect biologically meaningful changes at the cellular or microstructural level [8].

Among the various radiomic feature classes, first-order statistics describe the distribution of voxel intensities within a defined region of interest, without accounting for spatial relationships. Entropy, a first-order metric, quantifies the degree of randomness or unpredictability in voxel intensity values, serving as a marker of global heterogeneity. Increased entropy has been associated with disordered tissue architecture, necrosis, or infiltrative processes in several organ systems [9].

Second-order features, such as those derived from gray-level co-occurrence matrices (GLCM), extend this analysis by incorporating spatial dependencies between adjacent voxels. These metrics assess how frequently pairs of intensity values occur in specific spatial arrangements, thereby characterizing patterns of texture, granularity, and local disorganization. Metrics like GLCM entropy reflect not just global heterogeneity, but also the complexity of regional pixel interactions—features that may correspond to histological abnormalities such as lobular distortion, inflammatory infiltration, or early fibrotic remodeling [10].

In hepatic imaging, radiomic texture features have shown increasing utility in the assessment of chronic liver disease, including non-alcoholic steatohepatitis, fibrosis, and cirrhosis. Studies have demonstrated correlations between radiomic parameters and histopathologic markers of inflammation, collagen deposition, and architectural disruption. These findings suggest that radiomics may serve as a non-invasive surrogate for microscopic liver injury, especially in early disease stages when standard imaging remains inconclusive. Applying these principles to acute liver involvement in systemic viral infections such as COVID-19 may open new pathways for detecting and monitoring parenchymal damage beyond the capabilities of conventional attenuation-based techniques [11].

Given the frequent overlap between hepatic steatosis and inflammation in patients with SARS-CoV-2 infection, radiomic entropy features may provide enhanced sensitivity in detecting subclinical or persistent parenchymal injury that remains undetectable by conventional imaging metrics such as attenuation values or liver-to-spleen ratios. Inflammatory infiltration and cellular disorganization can increase tissue heterogeneity even in the absence of gross density changes, making entropy a potential surrogate marker for microscopic liver stress. This is particularly relevant in scenarios where steatosis may mask superimposed inflammatory damage, leading to underestimation of hepatic involvement. A longitudinal imaging approach that incorporates radiomic entropy, serial hepatic attenuation measurements, and liver-to-spleen ratio dynamics could yield a more comprehensive, non-invasive framework for characterizing liver injury throughout the course of COVID-19. By capturing both the structural disarray associated with acute inflammation and the evolving density patterns suggestive of steatosis or fibrotic transition, such a multi-parametric model may improve diagnostic accuracy and facilitate risk stratification. These tools may also have prognostic implications for long-term liver health in post-infectious states, particularly in patients with predisposing metabolic or hepatic conditions [12].

The aim of this study was to evaluate whether CT-based radiomic entropy can detect hepatic parenchymal alterations associated with inflammatory injury in patients with COVID-19, which are not appreciable by conventional CT assessment, and to analyze their relationship with liver enzyme elevations and follow-up imaging

## 2. Materials and Methods

### 2.1. Study Design and Population

This retrospective observational study included 41 adult patients with RT PCR confirmed moderate or severe COVID-19 pneumonia who underwent thoracoabdominal non contrast CT during the acute phase and had at least one follow-up scan between three and twelve months later. Patients were included based on the availability of diagnostic quality CT scans in DICOM format and complete clinical documentation. All datasets were anonymized using DicomCleaner build version: 2025-02-20, and each case was assigned a unique numerical identifier to ensure consistent data handling throughout the study.

### 2.2. Imaging Acquisition and Processing

All CT scans were performed on the same scanner (BrightSpeed Elite, GE Healthcare, Milwaukee, WI, USA) using a standardized non-contrast thoracoabdominal protocol at 120 kVp with automatic tube current modulation. Dose levels were within the standard range for chest–abdomen examinations. Images were reconstructed with a 512 × 512 matrix, in-plane pixel spacing of approximately 0.7–0.9 mm (depending on field of view), and 5 mm slice thickness. Intensities were expressed in standardized Hounsfield units (HU). The standard clinical protocol of the scanner was applied, including automatic corrections (beam hardening, scatter correction, water calibration) and a standard reconstruction kernel. Acquisition parameters were identical across acute and follow-up scans, ensuring reproducibility.

Image analysis was performed using 3D Slicer (version 5.6.2, https://www.slicer.org, accessed on 4 February 2025). Liver segmentation was conducted with the RVX Liver Segmentation module which applies a deep learning model trained to delineate hepatic anatomy in CT or MRI images. Each automated segmentation was visually inspected and manually refined as needed to ensure complete inclusion of the liver parenchyma while excluding major vessels and adjacent structures. A spherical region of interest was manually placed within the splenic parenchyma avoiding vascular and capsular areas to obtain reference attenuation values for calculating the liver to spleen attenuation ratio.

**Figure 1:**
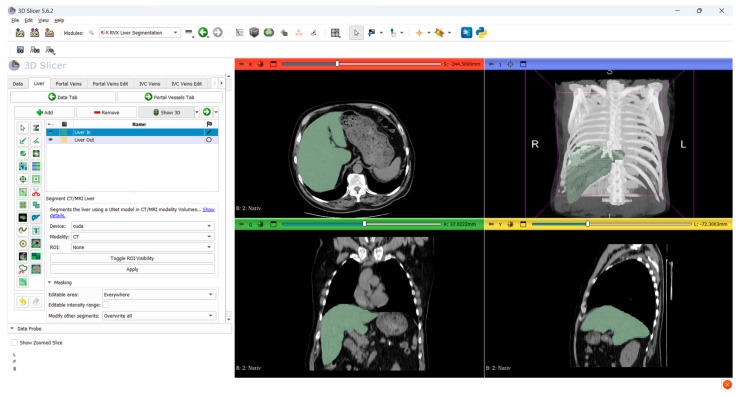
3D Slicer 5.6.2 software—RVX Liver Segmentation module.

After segmentation liver volume and mean hepatic attenuation expressed in Hounsfield units were extracted from the entire segmented liver using the Segment Statistics module. All measurements were based on DICOM images with an axial slice thickness of five millimeters. Radiomic analysis was subsequently performed using the Revision: 8426cdf extension integrated within 3D Slicer. From the full liver segmentation, two entropy-based features were selected a priori: first-order entropy and gray-level co-occurrence matrix (GLCM) entropy. First-order entropy quantifies the global randomness of voxel intensity distribution, whereas GLCM entropy reflects spatial disorder in gray-level co-occurrence. These complementary features were chosen for their biological plausibility in capturing hepatic heterogeneity and for their relative robustness to 5 mm slice reconstructions, where higher-order texture metrics are more susceptible to partial-volume and kernel effects (Figure 1).

All CT scans were evaluated independently by two board-certified radiologists, each with over five years of experience in thoracic imaging. Discrepancies were resolved by consensus.

Pulmonary involvement was assessed on the acute phase CT scans using the 25-point CT severity score method. This score along with relevant clinical and laboratory data was integrated into a structured database. For each patient both absolute values and longitudinal changes were computed between the acute and follow up phases including differences in liver volume hepatic attenuation first order entropy and joint entropy [13].

### 2.3. Histopathologic Correlation

Three full-body autopsies were conducted under strict biosafety conditions in the COVID-19-designated area of the County Clinical Emergency Hospital morgue. All patients had tested positive for SARS-CoV-2 by RT-qPCR at admission and died from confirmed severe pneumonia. Postmortem examinations included systematic macroscopic assessment and tissue sampling from multiple organs, followed by fixation in 10% neutral buffered formalin, paraffin embedding, and routine hematoxylin and eosin staining, in accordance with protocols previously used by our group.

These patients had also undergone native non-contrast CT at admission, allowing for direct correlation between hepatic imaging parameters—such as attenuation and entropy—and histopathologic findings, including steatosis, inflammatory cell infiltration, sinusoidal congestion, and hepatocellular degeneration

### 2.4. Statistical Analysis

Imaging and biochemical data were collected for all 41 patients, including hepatic volume, attenuation values for the liver and spleen, liver to spleen attenuation ratio, and radiomic features extracted during both the acute and follow up phases. Biochemical parameters reflected liver function and systemic inflammatory status. Volumetric, densitometric, and texture-based features were analyzed in relation to laboratory markers to investigate potential associations between hepatic imaging findings and biochemical evidence of liver injury. Liver injury was defined as an increase in aminotransferases, with AST or ALT values above the upper limit of normal (>40 U/L). Laboratory results were obtained at hospital admission, within the same time frame as the baseline CT examination, to ensure direct comparability between imaging and biochemical data (Table 1).

Paired differences between acute and follow-up imaging were assessed using the Wilcoxon signed-rank test. Comparisons between patients with and without hepatic injury were performed using the Mann–Whitney U test. Correlations between radiomic features and biochemical or clinical variables were examined using Spearman’s rank correlation coefficient. All statistical tests were two-tailed, with a significance threshold set at *p* < 0.05. Analyses were conducted using IBM SPSS Statistics version 26.

To address the risk of overfitting, internal validation was performed using 5-fold cross-validation and 1000-sample bootstrap resampling. Logistic regression models incorporating first-order and GLCM entropy were tested for both acute and follow-up datasets. Discrimination was summarized using mean cross-validated AUC and bootstrap AUC with 95% confidence intervals. In addition, false discovery rate (Benjamini–Hochberg) correction was applied to reduce the probability of false positive results.

## 3. Results

### 3.1. Radiomic Entropy Differentiates Hepatic Injury

Radiomic analysis demonstrated that both first-order entropy and GLCM entropy values were significantly elevated in patients with hepatic injury during the acute phase. Specifically, the mean first-order entropy was 1.63 in the injury group compared to 1.48 in the non-injury group (*p* = 0.019), while GLCM entropy values were 3.12 versus 2.83, respectively (*p* = 0.013). These results indicate increased hepatic parenchymal heterogeneity in affected individuals, likely reflecting inflammation-driven microstructural disorganization and early fibrotic processes.

ROC analysis showed that first-order entropy achieved an AUC of 0.703 (optimal threshold 1.564, sensitivity 66.7 percent, specificity 76.5 percent). GLCM entropy achieved an AUC of 0.708 (optimal threshold 3.068, sensitivity 58.3 percent, specificity 94.1 percent). Combining entropy with CT severity score improved discrimination to ~0.75 in cross-validation, suggesting added predictive value from integrating lung and liver imaging markers. Internal validation confirmed the stability of the entropy-based models. In the acute phase, first-order entropy alone achieved a cross-validated AUC of ~0.73 (bootstrap mean ~0.71, 95% CI 0.53–0.86). Adding AST and ALT biomarkers provided a modest additional improvement. Comparable results were obtained for follow-up and Δ models, with the combined Δ entropy + Δ AST/ALT model achieving the highest performance. In multivariable sensitivity analyses adjusting for age, sex, and BMI/nutritional status, effect estimates and AUC values remained consistent, suggesting that confounding did not substantially alter the associations. The full list of validation results is available in the Supplementary Materials.

### 3.2. Conventional CT Parameters Show Limited Discrimination

Conventional CT-derived parameters such as hepatic density, liver-to-spleen attenuation ratio (H/S), and liver volume were analyzed. In the acute phase, mean hepatic density was 53.8 HU in the injury group versus 48.7 HU in the non-injury group (*p* = 0.087). The H/S ratio showed a similar trend, with values of 0.97 and 0.89, respectively (*p* = 0.276). Hepatic volume did not differ significantly between groups.

### 3.3. Longitudinal Changes in CT Metrics

At follow-up, hepatic density was comparable between groups (52.2 vs. 52.8 HU, *p* = 0.856), with a relative increase of +10.0% in the non-injury group and a slight decline (−1.8%) in the injury group (*p* = 0.151). The H/S ratio increased in both groups, with a relative change of +5.4% versus +15.3% (*p* = 0.226), while liver volume showed minimal variation, decreasing by −2.0% in the injury group and −3.3% in the non-injury group (*p* = 0.779). No correlation was found between GLCM entropy and percentage change in hepatic volume (r = −0.015, *p* = 0.926), indicating that texture metrics reflect microarchitectural changes distinct from macroscopic volumetric alterations.

### 3.4. Entropy Correlates with Hepatic Density at Follow-Up

Correlation analyses showed that acute-phase entropy values were inversely associated with hepatic density at follow-up. GLCM entropy showed a moderate negative correlation (r = −0.385, *p* = 0.013), and first-order entropy followed the same pattern (r = −0.346, *p* = 0.027). These findings suggest that higher heterogeneity during acute illness may predict reduced parenchymal attenuation later on. However, in the acute phase, no significant correlation was observed between entropy and hepatic density (GLCM: r = −0.116, *p* = 0.469; first-order: r = −0.104, *p* = 0.518).

### 3.5. Liver Volumetry

Liver volumetry was evaluated in both the acute and follow-up phases. No significant correlations were found between hepatic volume and aminotransferases (AST, ALT) in either phase. In contrast, liver volume correlated negatively with hepatic density, both in the acute phase (r = –0.37, *p* = 0.018) and at follow-up (r = –0.48, *p* = 0.002). A positive correlation was also observed between liver volume at follow-up and first-order entropy (r = 0.40, *p* = 0.009), indicating that patients with larger liver volumes tended to show higher parenchymal heterogeneity. No significant correlations were observed between hepatic volume and GLCM entropy. These findings suggest that volumetric enlargement is more closely associated with fat-related attenuation loss and residual tissue heterogeneity rather than acute biochemical cytolysis.

### 3.6. Associations with Biochemical Markers

Entropy metrics also showed meaningful associations with markers of systemic inflammation and liver stress. First-order entropy was significantly correlated with serum ferritin (r = 0.47, *p* = 0.0017) and showed a borderline relationship with LDH (r = 0.30, *p* = 0.056). GLCM entropy correlated significantly with ferritin (r = 0.43, *p* = 0.0054). No relevant associations were found between entropy values and CRP, D-dimer, or CPK levels.

### 3.7. Histopathological Correlation

**Figure 2:**
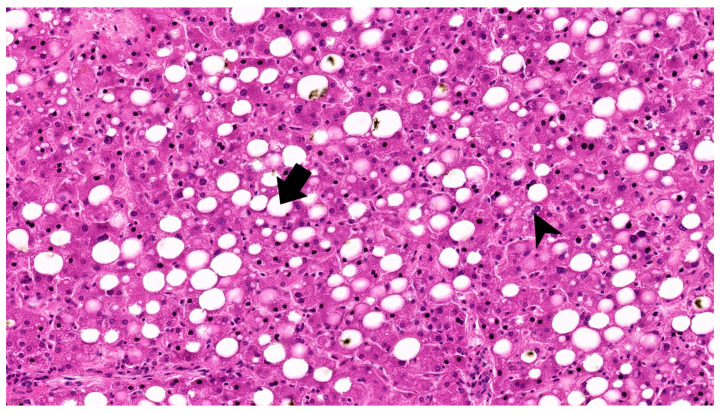
Case 1 Hematoxylin-Eosin (424×): Severe macro and microvesicular steatosis (arrow) with numerous bi or trinucleated hepatocytes (arrowhead).

CT-derived entropy values corresponded closely with the severity of microscopic liver injury. The case with the highest first-order (1.86) and GLCM entropy (3.51), also presenting elevated transaminases (AST 60, ALT 66), showed severe histologic alterations, including both macrovesicular and microvesicular steatosis, sinusoidal congestion, and a high number of bi- and trinucleated hepatocytes (Figure 2).

**Figure 3:**
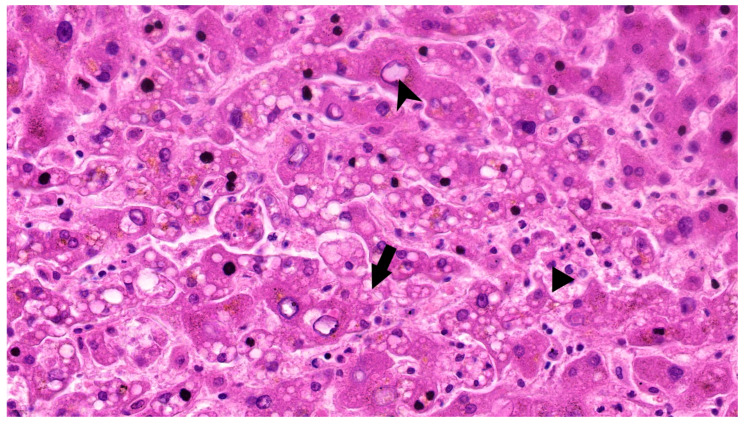
Case 2 Hematoxylin-Eosin (882×): Hepatocytes displaying multiple small and clear vacuoles (microvesicular steatosis) (arrow) and some glycogenated nuclei (arrowhead). Scattered neutrophils in capillary lumen and within Disse space (triangle).

Another case, with intermediate entropy (first-order 1.55, GLCM 2.80) and moderate transaminase elevation (AST 49, ALT 53), demonstrated histological features suggestive of moderate injury, such as mixed steatosis and vascular nuclear irregularity (Figure 3). 

**Figure 4:**
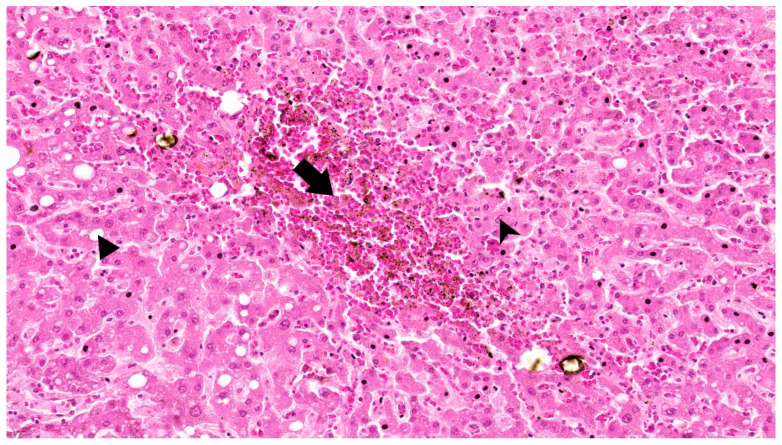
Case 3 Hematoxylin-Eosin (492×): Liver tissue presenting chronic passive congestion (arrow) and multiple binucleated hepatocytes or with enlarged nuclei (arrowhead) alongside areas of hepatic steatosis (triangle).

In contrast, the third case, with the lowest entropy (first-order 1.49, GLCM 2.83) and mild biochemical abnormalitie, exhibited only minimal histologic findings, such as microvesicular steatosis and scattered neutrophils. These comparisons support the utility of radiomic entropy as a non-invasive marker of liver parenchymal stress and architectural disorganization (Figure 4).

Overall, morphological modifications in liver histology in SARS-CoV-2 positive patients are characterized by steatosis, hepatocellular degeneration, inflammation, and vascular dysfunction.

### 3.8. Correlation Between Pulmonary Severity and Hepatic Entropy

Analysis revealed a modest but statistically significant association between pulmonary involvement and hepatic parenchymal heterogeneity during the acute phase. The CT severity score was positively correlated with first-order entropy (Spearman r = 0.332, *p* = 0.033), suggesting that patients with more severe lung injury tended to exhibit increased hepatic texture irregularity. GLCM entropy showed a similar trend (r = 0.283, *p* = 0.071), although this did not reach the conventional threshold for statistical significance. These findings may reflect a shared inflammatory mechanism driving both pulmonary and hepatic tissue disorganization in acute SARS-CoV-2 infection.

## 4. Discussion

Our results are consistent with and extend previous evidence indicating that hepatic injury in COVID-19 is not limited to metabolic or vascular mechanisms but also involves early inflammatory processes and structural disorganization detectable on imaging [14]. In our cohort, patients with biochemical evidence of liver injury demonstrated significantly greater hepatic parenchymal heterogeneity, as quantified by first-order entropy and GLCM entropy. Specifically, first-order entropy was higher in the injury group compared to the non-injury group (mean 1.63 versus 1.48, *p* = 0.019), as was GLCM entropy (3.12 versus 2.83, *p* = 0.013). These radiomic patterns likely reflect microarchitectural disruption induced by inflammatory or cytotoxic stress, capturing tissue-level changes that may precede conventional imaging abnormalities.

Furthermore, patients with elevated acute-phase entropy demonstrated lower hepatic density at follow-up, indicating a possible evolution toward steatosis. Entropy measured during the acute phase was inversely correlated with hepatic attenuation at follow-up (GLCM: r = −0.385, *p* = 0.013; first-order: r = −0.346, *p* = 0.027), suggesting that these metrics might anticipate the development or progression of fat accumulation. This is consistent with recent clinical studies reporting new-onset hepatic steatosis after SARS-CoV-2 infection, with observed prevalence rates between 25 and 40 percent [15].

This analysis focused intentionally on first-order entropy and GLCM entropy. These two metrics provide complementary insights into hepatic heterogeneity: first-order entropy measures the global randomness of voxel intensities, while GLCM entropy quantifies spatial disorder in gray-level co-occurrence. Both features are widely considered robust and interpretable radiomic descriptors. Given that all CT examinations were reconstructed at 5 mm slice thickness, higher-order texture metrics are more vulnerable to partial-volume and reconstruction-kernel effects, which may reduce reproducibility in small datasets. By restricting the evaluation to two biologically plausible and relatively stable features, this study aimed to minimize the risk of overfitting while retaining sensitivity to parenchymal changes. Future research with larger cohorts and thinner reconstructions may extend the feature set and build more complex predictive models

Other authors have shown that CT-based liver attenuation is reduced in patients with COVID-19 compared to controls, while the liver-to-spleen attenuation ratio has been used to stratify steatosis risk in this population [16]. However, traditional attenuation values may fail to detect early parenchymal changes in cases where inflammation coexists with steatosis. Radiomic features like entropy may therefore serve as sensitive markers of hepatic stress that are not confounded by fatty infiltration.

In our dataset, entropy metrics also correlated with systemic inflammatory markers. First-order entropy was significantly associated with serum ferritin (r = 0.47, *p* = 0.0017) and showed a borderline correlation with LDH (r = 0.30, *p* = 0.056). These relationships are consistent with prior findings that link ferritin to liver injury and disease severity in COVID-19. GLCM entropy likewise showed significant correlation with ferritin (r = 0.43, *p* = 0.0054), reinforcing its potential as a surrogate marker of tissue-level inflammation or cellular turnover.

Some patients exhibited very low hepatic attenuation values and abnormal H/S ratios. These were not considered artifacts, as all measurements were obtained from whole-liver segmentations, which minimize sampling error and reflect mean parenchymal density. Such findings most likely reflect genuine biological variation. In our cohort, some patients had pre-existing hepatic steatosis, while others appeared to develop steatosis during or after COVID-19, consistent with the report by Medeiros et al., who demonstrated a higher frequency of CT-detected steatosis among COVID-19 patients regardless of metabolic risk factors. This clinical heterogeneity underscores the limitations of mean attenuation values and further supports the role of entropy analysis in capturing parenchymal irregularity beyond HU averages [17].

Histopathological data available for a small subset of cases further supported the imaging findings. Increased entropy was observed in cases that demonstrated both macrovesicular and microvesicular steatosis, hepatocyte binucleation, nuclear irregularity, and inflammatory infiltration. In contrast, lower entropy values were found in livers with minimal cellular atypia or inflammation. These microscopic features are consistent with previously described hepatic alterations in COVID-19, including sinusoidal congestion, inflammatory infiltrates, and hepatocellular degeneration.

Several imaging studies support the hypothesis that liver injury during or after COVID-19 may manifest as either inflammatory or metabolic insult. For example, a large retrospective analysis reported significantly increased odds of CT-detected steatosis in post-COVID patients, independent of body mass index or metabolic status. Others have demonstrated the potential of non-contrast CT radiomics to identify early fibrotic or inflammatory hepatic changes in chronic liver disease, suggesting applicability in post-infection contexts [18].

Taken together, these findings highlight the potential utility of radiomic entropy in characterizing hepatic responses to systemic viral infections. Entropy may detect parenchymal stress that precedes steatosis, or alternatively, reveal inflammatory heterogeneity hidden by fat accumulation. Although further prospective validation is needed, radiomics may serve as a complementary approach for liver risk stratification in post-COVID populations.

The diagnostic performance observed in this study (AUC ~0.7) indicates only moderate discriminative ability. While not sufficient for clinical application, this result nevertheless supports the hypothesis that entropy reflects inflammatory changes in the liver parenchyma. Comparable studies have reported variable outcomes: Yoo et al. showed that radiomic features from non-contrast CT could predict liver fibrosis with an AUC of 0.78, whereas Wang et al. reported an AUC of 0.87 for cirrhosis prediction in HBV-infected patients. By contrast, Yoo and Parlak also reported inconclusive or inconsistent correlations between CT-based hepatic metrics and biochemical markers, underscoring the need for caution in interpretation. Attenuation-based studies in COVID-19 (e.g., Abdi and Ghaznavi) have shown associations between HU values and liver injury, but radiomics may provide added sensitivity by quantifying heterogeneity beyond mean density [11,19,20].

In addition to radiomic features, liver volumetry was also explored in our cohort. Although no significant correlation was found between hepatic volume and biochemical markers (AST, ALT), volumetric enlargement was associated with lower attenuation and higher entropy at follow-up, suggesting a relationship with fat-related changes and residual parenchymal heterogeneity rather than acute cytolysis. These findings are in line with recent evidence highlighting the clinical relevance of liver volumetry in COVID-19 and metabolic conditions. Bronte et al. demonstrated that liver volume may serve as an indicator of post-infectious and metabolic remodeling, underscoring the need for future studies to combine volumetric and radiomic analysis for a more comprehensive assessment [21].

All imaging analyses in this study, including radiomic feature extraction, hepatic density measurement and liver volumetry, were conducted using 3D Slicer. This open-source software provides robust segmentation and quantitative analysis tools without licensing fees or institutional restrictions. Its modular structure and support for workflow customization allow researchers to adapt protocols to specific study requirements. The use of 3D Slicer facilitates reproducibility and transparency, and its application in multiple published studies on hepatic CT imaging further supports the validity of this approach.

This study was limited by the absence of external validation. Although internal validation with cross-validation and bootstrap resampling mitigates the risk of overfitting, the findings remain exploratory and require confirmation in larger prospective cohorts.

The retrospective design represents another limitation, as enrollment was based on the availability of both baseline and follow-up CT scans together with laboratory data. While this ensured completeness of imaging–biomarker correlation, it does not equate to consecutive prospective enrollment and may reduce generalizability. Larger prospective cohorts are needed to validate these findings

## 5. Conclusions

This study demonstrates the potential of CT texture-based radiomic features in evaluating liver involvement in patients with SARS-CoV-2 infection. Conventional imaging metrics such as liver attenuation and volume did not consistently differentiate between patients with and without biochemical signs of hepatic injury. In contrast, radiomic parameters including first-order entropy and gray-level cooccurrence matrix entropy were significantly elevated in patients with transaminase elevations during the acute phase.

A significant inverse correlation was observed between entropy values measured during the acute stage and hepatic attenuation at follow up, suggesting that higher parenchymal heterogeneity may be linked to delayed resolution or the emergence of hepatic steatosis. The association between radiomic features and systemic inflammatory markers such as ferritin and lactate dehydrogenase supports their relevance as indicators of hepatocellular stress.

Histological evaluation in selected cases revealed changes such as steatosis, nuclear abnormalities, and portal inflammation, in line with imaging derived heterogeneity. These findings support the hypothesis that radiomic entropy may reflect both inflammatory and metabolic alterations within the liver parenchyma during COVID-19.

Larger prospective studies are required to confirm these results and to further explore the role of radiomics in detecting subclinical hepatic alterations following systemic infections.

## Figures and Tables

**Table 1:** Volumetric, Densitometric, and Texture-Based CT Features Alongside Laboratory Data.

Case ID	Hepatic Volume (Acute Phase), mL	Hepatic Volume (Follow-Up), mL	Mean Hepatic Density (Acute Phase), HU	Mean Hepatic Density (Follow-Up), HU	Mean Splenic Density (Acute Phase), HU	Mean Splenic Density (Follow-Up), HU	H/S (Acute Phase)	H/S (Follow-Up)	First-Order Entropy (Acute Phase)	GLCM Entropy (Acute Phase)	First-Order Entropy (Follow-Up)	GLCM Entropy Follow-Up)	Serum Ferritin, ng/mL	Serum LDH, U/L	AST, U/L	ALT, U/L	CRP, mg/L	D-Dimer, µg/mL	CPK, U/L	CT Severity Score (25-Point Scale)	Hepatic Injury (Yes = 1, No = 0)
1	1757.81	1748.3	60.99	60.36	53.87	49.34	1.13	1.22	1.47	2.78	1.33	2.64	773	532	31	111	68.74	674.37	124	20	1
2	2259.28	1978.97	46.37	52.77	52.82	48.08	0.88	1.1	1.56	3.07	1.43	2.72	519.6	398	33	55	140	290.28	66	12	1
3	1001.34	1147.72	59.01	57.82	50.4	49.81	1.17	1.16	1.58	2.95	1.32	3.16	1016.6	410	34	39	117.5	467.54	186	7	0
4	1880.64	1191.64	55.56	50.17	54.61	43.09	1.02	1.16	1.39	2.72	1.66	2.57	91.7	181	16	10	55.81	552.58	35	13	0
5	1909.28	1908.17	56.92	57.87	54.61	56.26	1.04	1.03	1.31	2.6	1.34	2.64	292.6	238	81	64	323.89	670.96	41	10	1
6	1914.85	1484.69	57.52	54.08	58.18	49.96	0.99	1.08	1.13	2.39	1.44	2.70	157.1	165	22	27	60.48	206.19	44	14	0
7	1673.48	1641.11	63.22	67.73	55.03	54.68	1.15	1.24	1.13	2.2	1.23	2.40	462.5	209	37	78	36.81	514.41	80	15	1
8	2315.51	2114.17	52.99	45.11	54.84	56.2	0.97	0.8	1.77	3.51	1.69	3.35	436.8	235	43	63	32.34	156.08	231	23	1
9	1329.74	1399.59	56.64	50.12	62.79	41.43	0.9	1.21	1.57	3.08	1.41	2.74	1978	250	65	162	79.13	877.29	90	15	1
10	2236.87	1655.17	31.37	61.88	55.78	56.21	0.56	1.1	1.57	3.05	1.63	3.14	1034	323	19	32	92.19	3212.97	51	20	0
11	1923.34	1728.08	53.42	57.23	60.69	58.74	0.88	0.97	1.49	2.88	1.59	3.02	1120	398	127	83	108.82	878.46	60	23	1
12	1334.51	1317.15	69.87	61.2	63.93	48.14	1.09	1.27	1.68	3.17	1.44	2.65	995.6	325	30	60	290.87	993.3	19	20	1
13	1815.88	1556.35	46.43	61.62	47.19	51.83	0.98	1.19	1.75	3.35	1.49	2.80	1272	373	57	43	58.41	423.05	435	17	1
14	934.93	1012.53	47.85	61.97	55.33	56.97	0.86	1.09	1.55	2.87	1.41	2.57	322.1	395	32	20	98.82	1329.88	63	17	0
15	1363.98	1348.25	49.49	50.93	56.9	55.75	0.87	0.91	1.72	3.25	1.72	3.23	743.3	407	91	75	63.95	932.68	392	12	1
16	2736.43	2654.28	44.21	25.39	66.68	52.17	0.66	0.49	2.06	4.01	1.93	3.77	1663	570	58	46	212.4	1441.14	62	16	1
17	1379.53	1412.94	64.74	49.36	52.44	51.77	1.23	0.95	2.02	3.78	1.50	2.81	1120	398	127	83	108.82	878.46	60	13	1
18	2622.16	2442.93	67.53	50.6	36.23	53.58	1.86	0.94	2.02	3.85	1.77	3.44	995.6	325	30	60	290.87	993.3	19	17	1
19	2022.81	2090.7	58.93	59.41	61.47	51.05	0.96	1.16	1.37	2.6	1.31	2.46	1272	373	57	43	58.41	423.05	435	23	1
20	2993.99	2234.12	34.1	35.77	58.07	58.41	0.59	0.61	1.56	3.02	1.51	2.98	322.1	395	32	20	98.82	1329.88	63	13	0
21	1821.66	2041.73	52.33	64.12	60.41	59.24	0.87	1.08	1.42	2.81	1.64	3.21	208.7	214	27	25	39.92	809.54	372	15	0
22	1659.23	1602.98	54.3	47.34	54.09	56.13	1	0.84	1.62	3.14	1.39	2.63	251.7	272	19	46	149.14	948.65	17	20	1
23	1902.63	1679.67	43.57	53.38	54.48	50.33	0.8	1.06	1.65	3.17	1.31	2.46	1038.7	399	29	47	24.7	200	191	11	1
24	2490.6	2218.64	35.26	30.76	58.81	60.14	0.6	0.51	1.48	2.83	1.52	2.82	633	360	33	23	141.2	972.51	950	20	0
25	1608.24	1748.43	53.33	62.04	53.29	59.97	1	1.03	1.39	2.61	1.74	3.31	136.7	271	26	23	0.47	356.3	105	15	0
26	2116.14	2104.48	43.2	65.71	54.11	59.48	0.8	1.1	1.48	2.79	1.41	2.78	432.2	398	60	90	47.55	1347.59	110	18	1
27	1838.23	2489.45	67.41	48.06	80.27	46.6	0.84	1.03	1.98	3.8	1.40	2.66	1119.9	224	75	119	5.47	326.99	110	17	1
28	1477.16	1394.07	55.15	60.21	55.66	57.67	0.99	1.04	1.35	2.54	1.24	2.46	715	412	26	30	133.76	1259.14	90	5	0
29	2019.17	2072.34	39.42	51.96	55.64	58.72	0.71	0.88	1.57	3	1.54	2.99	2000	608	63	2336	150.98	2050.28	182	7	1
30	1630.23	1233.25	54.35	53.19	55.58	48.09	0.98	1.11	1.42	2.7	1.40	2.75	504.6	245	44	88	19.55	484.8	539	20	1
31	1596.47	1332.85	55.44	60.57	46.94	55.06	1.18	1.1	1.55	2.9	1.26	2.31	1120	295	34	26	92.26	555.91	667	20	0
32	1896.05	1704.91	26.13	19.63	61.52	47.41	0.42	0.41	1.58	2.97	1.37	2.51	6604.7	501	55	32	221.44	1615.79	44	20	1
33	1171.2	1130.07	55.28	67.97	51.99	54.27	1.06	1.25	1.95	3.61	1.56	2.88	1500	361	76	124	46.6	442.7	212	15	1
34	1379.44	1374.85	49.43	51.08	55.98	47.18	0.88	1.08	1.62	3.01	1.54	3.01	625.1	216	26	26	38.54	846.79	131	15	0
35	1258.38	1321.51	50.47	51	57.95	54.47	0.87	0.94	1.69	3.21	1.51	2.99	633	360	33	23	141.2	972.51	950	12	0
36	1446.18	1440.12	64.95	58.05	54.72	56.43	1.19	1.03	1.18	2.29	1.13	2.22	1119.9	224	75	119	5.47	326.99	110	5	1
37	1615.04	1846	52.02	56.68	57.42	42.22	0.91	1.34	1.49	2.95	1.42	2.72	600	267	22	22	172.27	1884.54	450	18	0
38	1181.02	1128.47	45.38	49.26	48.78	45.23	0.93	1.09	1.51	2.86	1.40	2.63	202	245	24	21	4.1	447.09	97	13	0
39	2389.85	2864.49	44.67	30.48	49.26	45.47	0.91	0.67	1.39	2.73	1.57	3.09	320	379	20	24	39.43	637.65	61	11	0
40	1057.94	1149.58	49.62	60.49	59.04	55.57	0.84	1.09	1.47	2.69	1.04	2.02	1202	426	24	16	185.17	570.75	49	16	0
41	1570.9	1842.6	52.03	37.74	63.58	58.46	0.82	0.65	1.74	3.3	1.58	3.08	2000	432	89	87	115.35	1900.25	245	12	1

## Data Availability

The data presented in this study are available upon reasonable request from the corresponding author.

## Supplementary Materials

The following supporting information can be downloaded at: https://www.mdpi.com/article/10.3390/diagnostics15182364/s1, Table S1: Internal validation of entropy-based models 5-fold cross-validation and bootstrap resampling, *n* = 41.
