# Supplementary material for: Entropy-Based CT Radiomics as an Imaging Marker of Hepatic Injury in COVID-19
Source: Diagnostics (Basel). 2025 Sep 17;15(18):2364. doi: 10.3390/diagnostics15182364 (PMC12468892; doi:10.3390/diagnostics15182364)
Supplement: Supplementary file 1 [file diagnostics-15-02364-s001.zip › diagnostics-3839790-supplementary.pdf]

Table S1. Internal validation of entropy-based models 5-fold cross-validation and bootstrap resampling, $n = 41$.

Model	Predictors	N Used	Cross-Validated AUC (Mean ± SD)	Bootstrap Mean AUC	95% CI (Bootstrap)
**Acute: FO**	FO entropy (acute)	41	0.73 ± 0.08	0.71	0.53–0.86
**Acute: FO + AST/ALT**	FO entropy (acute) + AST + ALT	41	0.75 ± 0.07	0.73	0.55–0.88
**Acute: FO + AST/ALT + confounders**	FO entropy (acute) + AST + ALT + age, sex, BMI	39	0.76 ± 0.07	0.74	0.56–0.89
**Follow-up: FO**	FO entropy (follow-up)	41	0.72 ± 0.09	0.70	0.52–0.85
**Δ Model: FO**	Δ FO entropy (follow-up − acute)	41	0.71 ± 0.10	0.69	0.50–0.84
**Δ Model: FO + ΔAST/ΔALT**	Δ FO entropy + Δ AST + Δ ALT	41	0.74 ± 0.08	0.72	0.53–0.88

Confounders included: age, sex, BMI status. FO: First-order SD: Standard deviation AUC: Area under the curve CI: Confidence interval AST: Aspartate aminotransferase ALT: Alanine aminotransferase.
